# Global and regional extracellular volume and myocardial circumferential strain correlate significantly in a heterogeneous patient population

**DOI:** 10.1186/1532-429X-18-S1-P260

**Published:** 2016-01-27

**Authors:** Naveen Rajpurohit, Zhen Qian, Shizhen Liu, Robi Goswami, Ashish Kabra, Venkateshwar Polsani, Mani Vannan

**Affiliations:** Cardiovascular imaging, Piedmont Heart Institute, Atlanta, GA USA

## Background

Myocardial extracellular volume (ECV) quantification by cardiovascular magnetic resonance (CMR) T1 mapping technique has shown great promises in detecting diffuse fibrosis and interstitial diseases of the myocardium. In hypertensive and left ventricle hypertrophy (LVH) patients, increased ECV has been found to be associated with impaired myocardial strain, which is an early indicator of cardiac dysfunction. We hypothesized that in a heterogeneous patient population, ECV was significantly associated with myocardial strain, on the global and the more localized segmental bases.

## Methods

Prospectively, 37 consecutive heterogeneous patients (Age 59 ± 15; 78% male) underwent ECV and grid-tagged CMR imaging studies on a Siemens 1.5T Avanto, among whom 12 were normal (group 1), 12 had coronary artery disease (group 2), 4 had non-ischemic cardiomyopathy (group 3) and 5 had hypertrophic cardiomyopathy (group 4). T1 was measured pre-contrast and 10 minutes after contrast administration at the mid short axis (SA) using the MOLLI technique. The pulse sequence parameters included: flip angle 35°, FOV 400 × 300 mm, resolution 1.6 × 1.6 mm, and slice thickness of 8 mm. ECV was calculated based on the mean and segmental T1 values and adjusted for the hematocrit. The grid-tagged images were acquired at the same mid SA locations. The pulse sequence parameters included: TE 3.4 ms, TR 37 ms, flip angle 15°, FOV 400 × 300 mm, resolution 2.1 × 2.1 mm, and slice thickness of 8 mm. Myocardial strain was calculated using a grid-tracking-based tagged MRI analysis software. Pearson correlation coefficient was calculated for linear association analysis. Analysis of variance (ANOVA) was used to determine significant differences between the mean ECV and mean global circumferential strain of the 4 groups. p < 0.05 was considered significant.

## Results

**Figure 1:**
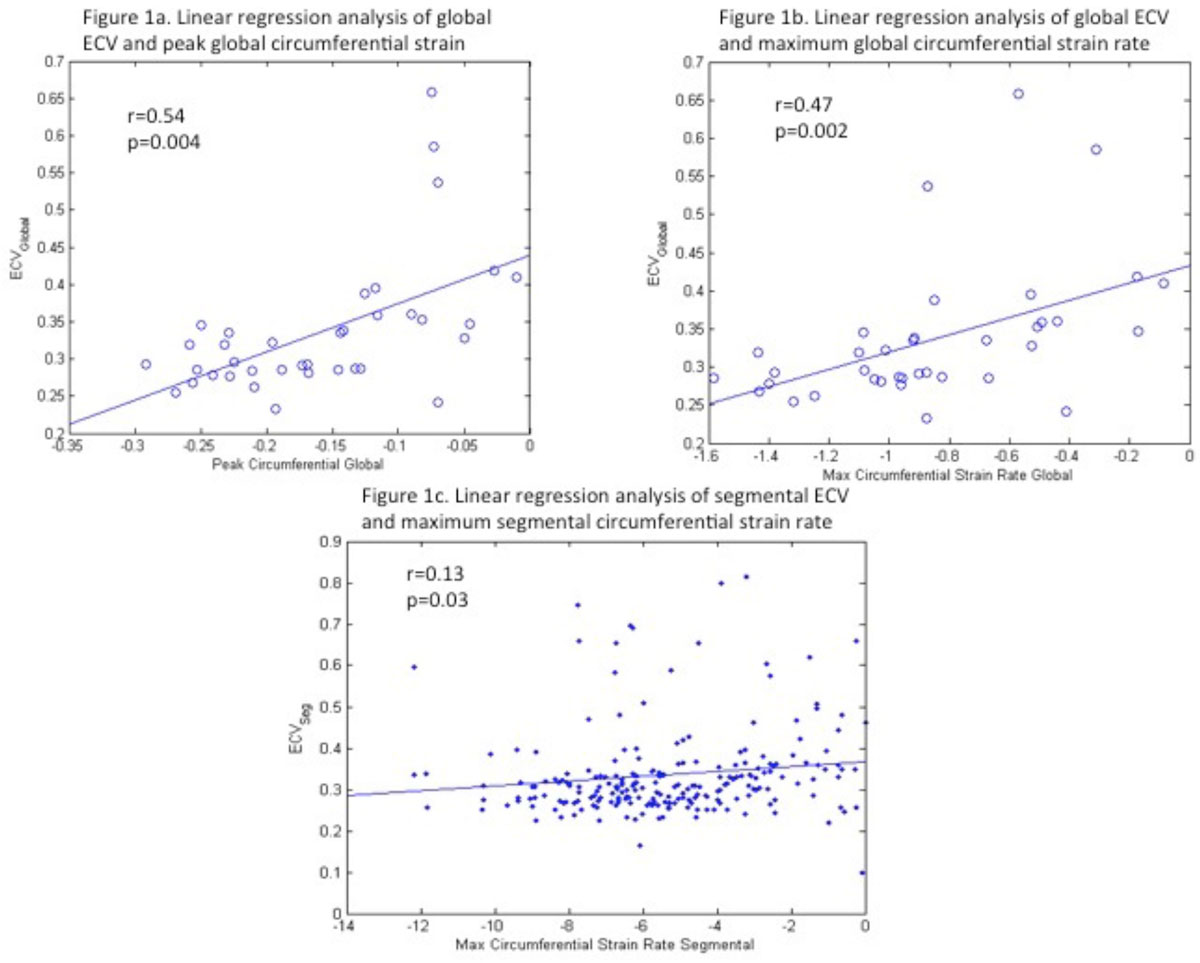
**Figures 1a-b show linear correlation between global ECV and peak circumferential global strain (figure 1a) and maximum circumferential global strain rate (figure 1b)**. Figure 1c shows the correlation between segmental ECV and maximum segmental circumferential strain rate.

As shown in Figure 1a-b global ECV had significant correlation with the peak global circumferential strain and maximal global circumferential strain rate(r = 0.54 and p =0.004 and r = 0.47 and p = 0.002 respectively). The segmental ECV also correlated significantly with the maximum segmental circumferential strain rate (r = 0.14 and p = 0.03, figure 1c).

**Figure 2:**
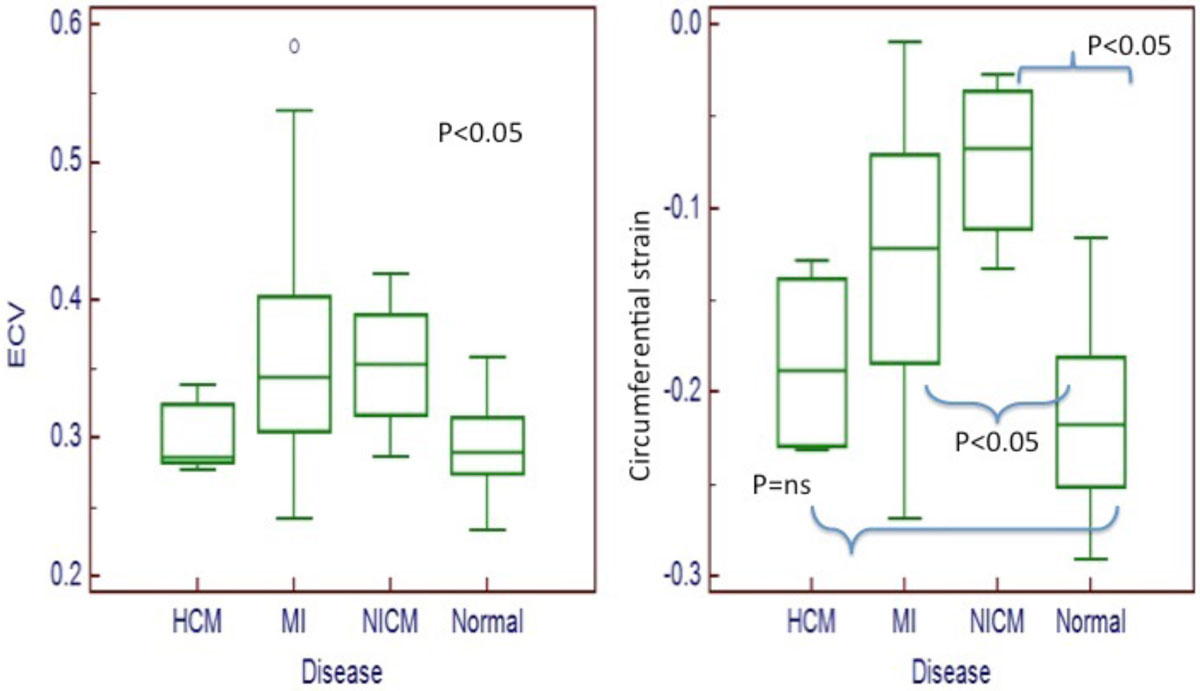
**shows the differences between the mean ECV and mean global circumferential strain in the 4 groups of patients**.

As shown in Figure 2, ECV was a better discriminator than global circumferential strain between the four groups of patients. Global circumferential strain was significantly different between the groups 1, 2, 3 but not between groups 1 (normal) and 4 (hypertrophic cardiomyopathy). Global ECV was significantly different between all the groups. The main limitation of this data is the small sample size.

## Conclusions

In a heterogeneous group of patient population, global and segmental myocardial ECV obtained from CMR T1 mapping images significantly correlated with the global and segmental myocardial circumferential strain. Global ECV was better than global circumferential strain at discriminating between normal and hypertrophic cardiomyopathy patients.

